# Do Biochemical Markers Reflect Parathyroid Adenoma Volume in Primary Hyperparathyroidism?

**DOI:** 10.3390/ijms27115034

**Published:** 2026-06-02

**Authors:** Şebnem Çimen, Nazlıcan Altındağ, Ergin Erginöz, Mehmet Zeki Buldanlı, Murat Özkara, Burak Uçaner

**Affiliations:** 1 Department of General Surgery, Akyurt State Hospital, 06750 Ankara, Türkiye; 2 Department of General Surgery, Gülhane Training and Research Hospital, University of Health Sciences, 06010 Ankara, Türkiye; naz.nazli.nazlican@gmail.com (N.A.); buldanli87@hotmail.com (M.Z.B.); opdrmozkara@gmail.com (M.Ö.); burakucaner@hotmail.com (B.U.); 3 Department of General Surgery, Memorial Sloan Kettering Cancer Center, New York, NY 10065, USA; eerginoz@ku.edu.tr

**Keywords:** primary hyperparathyroidism, parathyroid adenoma, adenoma volume, serum calcium, vitamin D

## Abstract

The relationship between biochemical severity parameters and adenoma burden in primary hyperparathyroidism (PHPT) has been widely studied, although some variability across studies persists. This relationship is clinically relevant for improving disease assessment and guiding surgical decision-making. This study aimed to investigate the association between preoperative biochemical parameters and both ultrasonographic and histopathological adenoma volume. A total of 526 patients with histopathologically confirmed solitary parathyroid adenoma who underwent surgery between January 2017 and December 2024 were retrospectively analyzed. Preoperative corrected serum calcium, parathyroid hormone (PTH), 25-OH vitamin D, phosphorus, albumin, and alkaline phosphatase (ALP) levels were recorded. Adenoma volume was calculated using ultrasonographic measurements and histopathological dimensions, and correlations were evaluated with appropriate statistical methods. Corrected serum calcium showed a significant positive correlation with histopathological adenoma volume (r = 0.249, *p* < 0.001; ρ = 0.340, *p* < 0.001), while its relationship with ultrasonographic volume was weaker. Ultrasonographic and histopathological volumes were strongly correlated (r = 0.68, *p* < 0.001). Vitamin D demonstrated a significant negative correlation with both volume measurements. PTH showed no significant association with either ultrasonographic or histopathological adenoma volume. No significant associations were found for phosphorus or albumin, and ALP showed weak correlations. Corrected serum calcium was associated with adenoma volume, particularly when assessed histopathologically, suggesting that serum calcium may partially reflect adenoma burden in PHPT, although the observed associations were modest.

## 1. Introduction

Primary hyperparathyroidism (PHPT) is a common endocrine disorder characterized by increased secretion of parathyroid hormone (PTH) and consequent hypercalcemia. In the majority of cases, the underlying cause is a single parathyroid adenoma, accounting for approximately 80–90% of PHPT cases [1,2]. Although PHPT can now be diagnosed at an earlier stage due to routine biochemical testing, it may still lead to significant complications affecting the renal and skeletal systems [2,3].

The relationship between adenoma size and disease severity is clinically important for both evaluation and surgical planning. In theory, larger adenomas contain a greater mass of chief cells, which may result in higher levels of PTH and serum calcium [3]. Therefore, defining the association between adenoma volume and biochemical parameters may help better characterize disease phenotype and optimize surgical strategies.

The relationship between adenoma size and biochemical parameters has been investigated for decades. Early studies reported a positive correlation between adenoma weight or volume and serum calcium and PTH levels [4]. However, some series have shown that laboratory parameters do not reliably predict gland size and that the correlation may be weak [5].

More recent studies using pathological adenoma volume have demonstrated that larger adenomas show a significant positive correlation with preoperative PTH and calcium levels, whereas phosphorus and 25-OH vitamin D levels tend to decrease with increasing adenoma size [6]. Similarly, moderate-sized series from Türkiye have reported positive correlations between adenoma volume and PTH and calcium, and a negative correlation with vitamin D levels [7,8].

Vitamin D deficiency has been shown to influence the clinical and biochemical phenotype of PHPT and may be associated with more pronounced hypercalcemia [9]. Therefore, not only PTH and calcium but also metabolic parameters such as vitamin D and phosphorus should be evaluated in relation to adenoma volume.

Differences between imaging-based estimates of adenoma volume and histopathologically measured actual volume have also been discussed in the literature. Although a correlation between ultrasonographic and pathological volumes has been reported, variations in volume calculation methods may affect the results [10]. This suggests that discrepancies between imaging metrics and true tissue volume may influence the consistency of studies investigating biochemical correlations.

Taken together, these findings highlight the need for studies with larger sample sizes that evaluate both ultrasonographic and pathological adenoma volumes to better clarify their relationship with biochemical parameters, particularly given the variability in reported associations and differences in volume assessment methods.

This study aimed to investigate the associations between preoperative biochemical parameters, particularly corrected serum calcium and both ultrasonographic and histopathological adenoma volumes in patients undergoing surgery for primary hyperparathyroidism.

## 2. Results

A total of 526 patients with primary hyperparathyroidism were included in the study. The mean age was 52.44 ± 13.86 years, with 82.5% female (n = 434) and 17.5% male (n = 92). The mean body mass index (BMI) was 26.25 ± 1.02 kg/m^2^. The majority of patients were classified as ASA II (75.5%). The median Charlson Comorbidity Index was 2 (IQR 0–3), and a substantial proportion of patients had at least one comorbid condition (Table 1).

In the preoperative biochemical evaluation, mean serum calcium was 11.26 ± 0.72 mg/dL, albumin 4.21 ± 0.47 g/dL, PTH 198.49 ± 319.50 pg/mL, phosphorus 2.63 ± 0.82 mg/dL, 25-OH vitamin D 21.18 ± 11.65 ng/mL, and ALP 118.99 ± 42.58 U/L (Table 1).

**Figure 1:**
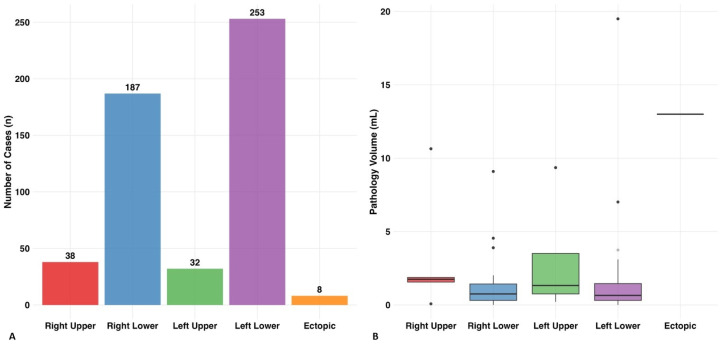
Distribution of Parathyroid Adenoma Localization and Volume. (**A**) Bar chart demonstrating the anatomical distribution of parathyroid adenomas. The most frequent localization was the left inferior gland, followed by the right inferior gland, while ectopic localization was rare. (**B**) Boxplot showing the distribution of pathological adenoma volumes across localization groups. The central line represents the median, the box indicates the interquartile range (IQR), the whiskers denote the range within 1.5× IQR, and circles represent outliers.

Regarding adenoma localization, the most common sites were the left inferior (n = 253) and right inferior (n = 187) glands; 38 cases were right superior, 32 left superior, and 8 ectopic (Figure 1). A significant difference in pathological volume distribution was observed among localization groups (*p* < 0.001).

**Figure 2:**
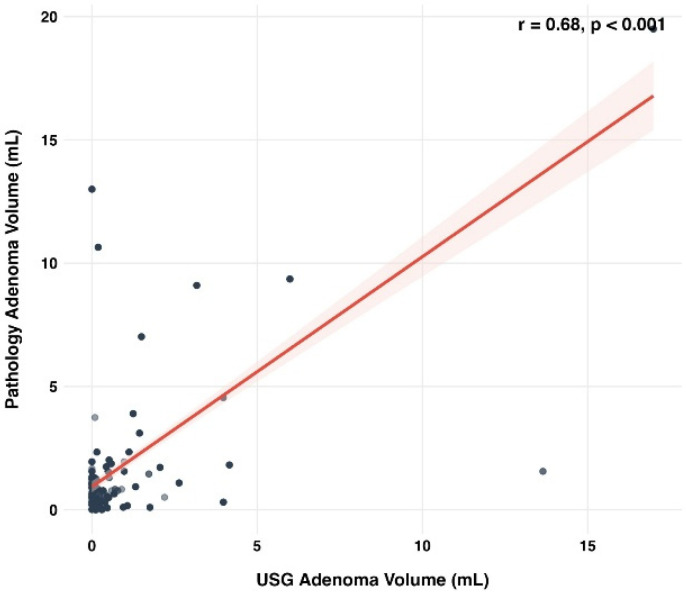
Correlation Between Ultrasonographic and Histopathological Adenoma Volume. Scatter plot illustrating the relationship between ultrasonographic (USG) and histopathological adenoma volume. A strong positive correlation was observed (ρ = 0.68, *p* < 0.001).

A strong and significant positive correlation was found between ultrasonographic (US) adenoma volume and histopathological adenoma volume (r = 0.68, *p* < 0.001) (Figure 2).

**Figure 3:**
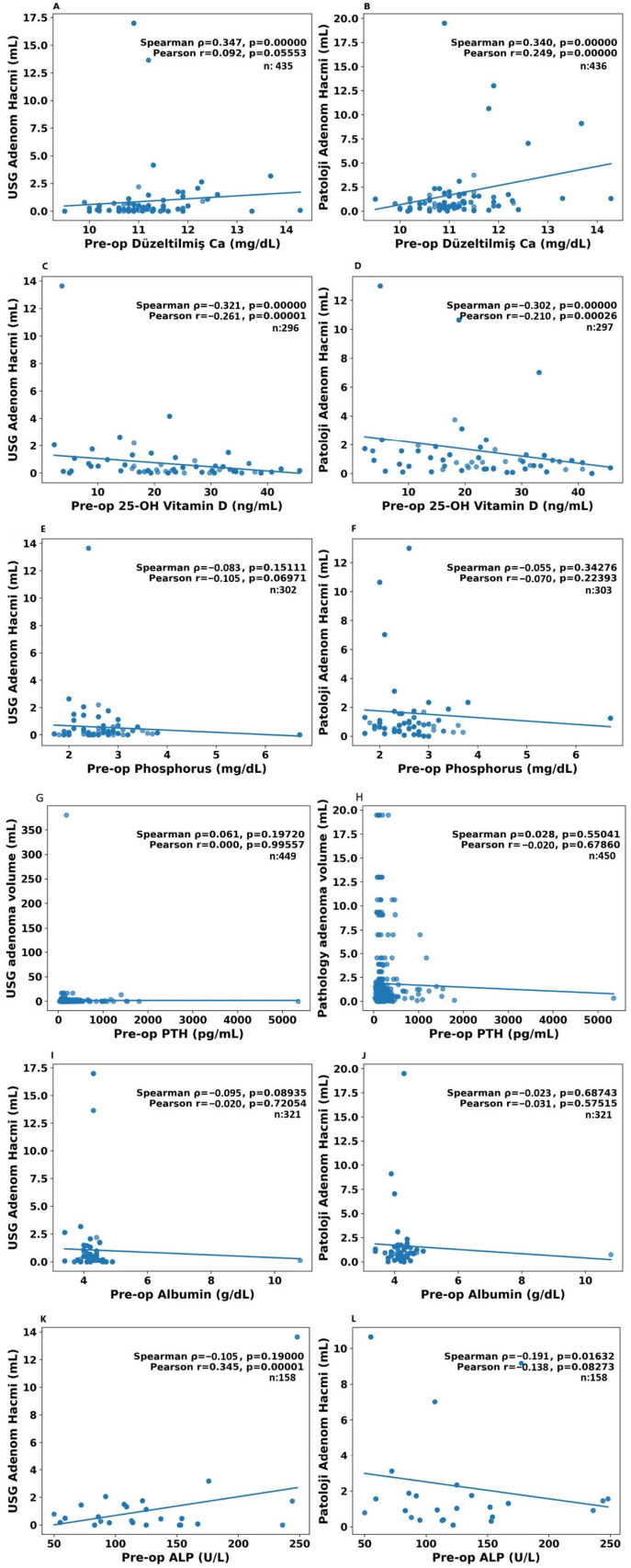
Correlation Between Preoperative Biochemical Parameters and Adenoma Volume. Scatter plots showing the association between preoperative biochemical parameters and adenoma volume measured by ultrasonography (USG) and histopathology: (**A**,**B**) corrected calcium, (**C**,**D**) 25-OH vitamin D, (**E**,**F**) phosphorus, (**G**,**H**) PTH, (**I**,**J**) albumin, and (**K**,**L**) ALP. Spearman (ρ) and Pearson (r) correlation coefficients are displayed within each panel. Corrected calcium was calculated as: Corrected Ca = Measured Ca + 0.8 × (4.0 − albumin). Statistical significance was defined as *p* < 0.05 (two-tailed). Abbreviations: USG, ultrasonography; PTH, parathyroid hormone; ALP, alkaline phosphatase.

A significant monotonic relationship was observed between preoperative corrected serum calcium and US adenoma volume (Spearman ρ = 0.347, *p* < 0.001), whereas the linear correlation was borderline (Pearson r = 0.092, *p* = 0.055; n = 435) (Figure 3A). In contrast, both Spearman (ρ = 0.340, *p* < 0.001) and Pearson (r = 0.249, *p* < 0.001; n = 436) analyses demonstrated a significant positive correlation between corrected calcium and histopathological volume (Figure 3B).

Preoperative 25-OH vitamin D levels showed a significant negative correlation with US volume (Spearman ρ = −0.321, *p* < 0.001; Pearson r = −0.261, *p* < 0.001; n = 296) (Figure 3C). A similar negative correlation was observed between vitamin D levels and histopathological volume (Spearman ρ = −0.302, *p* < 0.001; Pearson r = −0.210, *p* = 0.00026; n = 297) (Figure 3D).

No significant correlation was found between preoperative phosphorus levels and US volume (Spearman ρ = −0.083, *p* = 0.151; n = 302) or histopathological volume (Spearman ρ = −0.055, *p* = 0.343; n = 303) (Figure 3E,F).

Preoperative PTH levels were not significantly correlated with either US adenoma volume (Spearman ρ = 0.061, *p* = 0.1972; n = 449) or histopathological adenoma volume (Spearman ρ = 0.028, *p* = 0.5504; n = 450) (Figure 3G,H).

No significant correlation was identified between preoperative albumin levels and US volume (Spearman ρ = −0.095, *p* = 0.089; n = 321) or histopathological volume (Spearman ρ = −0.023, *p* = 0.687; n = 321) (Figure 3I,J).

While no significant relationship was observed between preoperative ALP levels and US volume (Spearman ρ = −0.105, *p* = 0.190; n = 158), a weak negative correlation was found between ALP and histopathological volume (Spearman ρ = −0.191, *p* = 0.016; n = 158) (Figure 3K,L).

In comparisons according to surgical indication groups, no significant difference was found in postoperative calcium levels (*p* = 0.205). However, postoperative PTH levels differed significantly between groups (*p* = 0.044). Adenoma volume calculated by US did not differ between indication groups (*p* = 0.516), whereas histopathological adenoma volume showed a significant difference (*p* < 0.001) (Table 2). Notably, patients with musculoskeletal symptoms had higher mean pathological volumes (2.73 ± 4.55 mL), while lower volumes were observed in the hypercalcemia group. No patients were operated on due to renal involvement (GFR < 60 mL/min).

**Figure 4:**
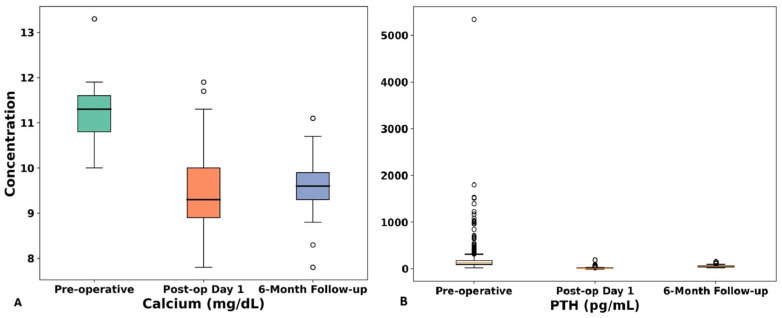
Perioperative Changes in Calcium and PTH. (**A**) Serum calcium (mg/dL) levels preoperatively, on postoperative day 1, and at 6 months, showing an early decline with sustained normocalcemia. (**B**) Serum PTH (pg/mL) levels demonstrate a marked postoperative decrease consistent with biochemical cure. Boxes indicate interquartile range (IQR); central line represents median; whiskers denote minimum–maximum values; circles represent outliers.

Postoperative biochemical evaluation demonstrated a marked decrease in serum calcium levels on postoperative day 1, with normocalcemia maintained at the 6-month follow-up (Figure 4A). Similarly, PTH levels showed a dramatic decline in the early postoperative period (Figure 4B).

Among 473 patients with available complication data within the first postoperative month, 80.8% (n = 382) experienced no complications. The most common complication was early hypocalcemia, observed in 67 patients (14.2%). Reoperation due to bleeding occurred in 7 patients (1.5%), vocal cord paralysis in 8 patients (1.7%), and hypoparathyroidism in 8 patients (1.7%). Surgical site infection was observed in only one patient (0.2%). No mortality was recorded.

Of the 67 patients who developed early hypocalcemia, 36 had 6-month follow-up data, and all showed normalization of serum calcium levels, with no cases of permanent hypocalcemia. Among 9 patients with corrected calcium < 8.5 mg/dL at 6 months, none belonged to the early hypocalcemia group, and they had not been coded as hypoparathyroidism in early complication records (Table 3).

The median follow-up duration was 8 months (IQR: 2–35 months). Recurrence was observed in 7 of 482 patients with available follow-up data (1.45%). The rate of persistent disease was low, and the overall biochemical cure rate exceeded 98% (Table 3).

## 3. Discussion

The relationship between biochemical severity and its morphological counterpart, gland volume, in primary hyperparathyroidism has been widely investigated, with generally consistent findings. In particular, the extent to which serum calcium reflects adenoma volume is clinically relevant for both understanding disease phenotype and guiding surgical planning. In this study, the association between corrected serum calcium and adenoma volume was systematically evaluated in a large cohort of patients with histopathologically confirmed solitary adenomas. The findings demonstrated a significant positive correlation between pathological adenoma volume and corrected serum calcium.

Previous studies have reported a positive association between adenoma size and serum calcium levels. Bindlish et al. demonstrated a significant correlation between adenoma weight and serum calcium [4]. More recently, Fiore et al. reported that adenomas larger than 2 mL were associated with higher preoperative calcium levels [6]. These findings suggest that hypercalcemia may become more pronounced as gland volume increases. In contrast, Mózes et al. reported that biochemical parameters do not reliably predict gland size and that the relationship between calcium and volume is inconsistent [5]. The significant correlation observed in the present study is consistent with studies supporting this relationship, while suggesting that previously conflicting results may largely stem from differences in sample size and methodology.

The borderline linear relationship between ultrasonographic volume and corrected calcium is noteworthy. Although a strong correlation was observed between ultrasonographic and pathological volumes, the associations between biochemical parameters and volume were modest. These findings suggest that imaging-based measurements and pathological assessment may capture different aspects of adenoma characteristics rather than one being superior to the other. Previous studies have demonstrated a generally strong correlation between ultrasonographically calculated volume—based on the ellipsoid formula—and pathological volume [10,11,12], although systematic differences between these methods have also been reported. In addition, some studies have found weak associations between ultrasonographic volume and biochemical parameters [13]. Taken together, these findings indicate that both imaging-based and histopathological measurements provide complementary information and should be interpreted within their respective clinical contexts.

The weak relationship between PTH levels and adenoma volume is consistent with the heterogeneous findings reported in the literature. While some studies have shown a positive correlation between PTH and adenoma weight [4], others have demonstrated that PTH levels do not reliably predict gland size and may show weak or no significant correlation [5]. In this study, PTH values showed a skewed distribution, and therefore non-parametric methods were preferred for correlation analyses. The absence of a significant relationship between PTH and histopathological volume suggests that circulating PTH levels may not consistently reflect gland volume in a linear manner. The pulsatile nature of PTH secretion and inter-individual biological variability may explain this discrepancy. In addition, serum calcium reflects the cumulative downstream physiological effect of PTH activity, whereas circulating PTH measurements may demonstrate greater biological fluctuation and may not proportionally reflect adenoma burden. This observation may also be explained by the concept of “secretory efficiency and capacity”, whereby larger adenomas, despite having greater cellular mass, may secrete less PTH per unit tissue compared to smaller glands. This may contribute to the weak or inconsistent relationship between circulating PTH levels and adenoma volume observed across studies [14,15].

The negative correlation between vitamin D levels and adenoma volume represents another important finding. Viccica et al. demonstrated that low 25-OH vitamin D levels are associated with a more severe biochemical phenotype [9]. Similarly, other studies have reported an inverse relationship between vitamin D levels and adenoma size [16]. The observed negative correlation between both ultrasonographic and histopathological volumes and vitamin D levels suggests that metabolic status may influence adenoma phenotype.

The lack of a significant relationship between phosphorus levels and adenoma volume is consistent with existing literature. Current guidelines suggest that phosphorus levels are primarily influenced by the renal effects of PTH and do not directly reflect gland volume [17]. Therefore, the absence of a significant association in this study can be considered an expected finding.

Albumin did not demonstrate a significant association with adenoma volume. This finding was expected, as serum albumin is not considered a direct marker of adenoma burden or disease severity in primary hyperparathyroidism. Albumin was evaluated primarily as part of the systematically assessed biochemical profile and because of its role in corrected calcium calculation.

The weak association between ALP levels and adenoma volume also aligns with previously reported heterogeneous results. Although some studies suggest that ALP may reflect disease severity, a strong and direct correlation with adenoma volume has not been consistently demonstrated [13,18]. These findings imply that bone turnover markers may reflect systemic effects rather than gland size. This may be explained by the fact that ALP reflects bone turnover activity, which is influenced by systemic metabolic effects of PTH rather than directly by adenoma size.

The observed difference in pathological volume according to adenoma localization suggests that anatomical factors may influence gland size. Previous studies have shown that inferior gland adenomas are more common than superior ones [19], which may be related to their longer embryological migration and greater positional variability [20]. In the present study, the predominance of inferior gland involvement may have practical implications, as in cases of inconclusive preoperative localization, prioritizing exploration of the inferior glands may increase the likelihood of identifying the adenoma, consistent with previous reports [19,20].

When surgical indication groups were examined, the finding of larger pathological volumes in patients with musculoskeletal symptoms suggests a possible association between gland size and clinical phenotype. Some studies have reported that larger adenomas are associated with more pronounced clinical presentations [5], whereas others have found no significant relationship between symptom severity and volume [13]. These findings indicate that the relationship between adenoma size and clinical phenotype is not deterministic.

The high postoperative biochemical cure rate (>98%) and low recurrence rate observed in this study are consistent with data reported from high-volume centers [17]. This supports the clinical reliability of the observed correlations.

This study has several methodological limitations. First, its retrospective and single-center design may introduce biases related to data completeness and recording quality. Multiglandular disease and parathyroid carcinoma were excluded; therefore, the findings primarily apply to solitary adenoma phenotypes and should be generalized to the broader PHPT spectrum with caution. Biochemical parameters were based on the last preoperative measurements, and temporal variability and preanalytical differences could not be controlled. In particular, the biological variability of PTH may have influenced correlation analyses. Ultrasonographic volume was calculated using the ellipsoid formula, and true three-dimensional volumetric analysis was not performed, which may limit the precision of imaging-based correlations. Furthermore, analyses were limited to correlation methods, and independent predictors were not evaluated using multivariable models; thus, potential confounding factors cannot be fully excluded. Finally, the heterogeneous follow-up duration may limit the assessment of long-term recurrence. Overall, these findings are descriptive in nature and should not be interpreted as predictive at the individual patient level.

## 4. Materials and Methods

### 4.1. Study Design and Ethical Approval

This study is a single-center, retrospective, observational cohort study evaluating patients operated on for primary hyperparathyroidism (PHPT). The research was conducted at a tertiary university hospital.

The study protocol was approved by the Non-Interventional Scientific Research Ethics Committee of the University of Health Sciences, Gülhane Training and Research Hospital on 8 May 2025 (approval number: 2025/86). The study was performed in accordance with the Declaration of Helsinki, and due to its retrospective design, the requirement for individual informed consent was waived by the ethics committee. All data were anonymized prior to analysis.

### 4.2. Study Population

Patients who underwent parathyroidectomy for PHPT between January 2017 and December 2024 were identified through the hospital information management system. Cases with histopathologically confirmed solitary parathyroid adenoma were included in the study.

Patients with multiglandular disease, parathyroid hyperplasia, parathyroid carcinoma, concomitant malignant thyroid pathology, or incomplete clinical, biochemical, or imaging data were excluded. These criteria were applied to establish a homogeneous “single adenoma” cohort.

### 4.3. Clinical and Laboratory Data

Demographic data (age, sex, body mass index, etc.), along with clinical information and laboratory results, were retrospectively obtained from the hospital electronic records and archived patient files. The Charlson Comorbidity Index and ASA classification were also recorded.

Preoperative biochemical parameters were defined as values measured within one month prior to surgery. The parameters included in the analysis were serum total calcium (mg/dL), serum albumin (g/dL), corrected calcium (mg/dL), intact parathyroid hormone (PTH) (pg/mL), 25-hydroxyvitamin D (ng/mL), serum phosphorus (mg/dL), and alkaline phosphatase (ALP) (U/L).

Serum total calcium levels were corrected for albumin concentration, and the corrected calcium value was calculated using the following formula:

Corrected Ca = Measured Ca + 0.8 × (4.0 − serum albumin)

Measurements corresponding to days on which parathyroid aspiration washout procedures had been performed were excluded to avoid potential analytical distortion. For each patient, the closest verified true serum preoperative PTH value prior to surgery was selected for analysis.

Cases with missing data were excluded from the relevant analyses, and the varying sample sizes (n) reflect data availability for each parameter.

### 4.4. Postoperative Assessment and Follow-Up Definitions

Postoperative biochemical evaluation was assessed in two phases: early and late periods.

The early postoperative period included laboratory measurements obtained within the first 24 h after surgery. Serum calcium (mg/dL) and PTH (pg/mL) levels were evaluated as early biochemical indicators of surgical success. Early hypocalcemia was also recorded during this period and was defined as a corrected serum calcium level < 8.5 mg/dL within the first 6 months.

The late postoperative period included outpatient follow-up evaluations starting from 6 months after surgery. Serum calcium and PTH levels measured during this period were assessed for persistent normocalcemia, persistent disease, or recurrence. Persistent hyperparathyroidism was defined as failure to achieve normocalcemia in the early postoperative period, whereas recurrence was defined as the reappearance of hypercalcemia after an initial period of normocalcemia. Cases with corrected calcium < 8.5 mg/dL at the sixth month were defined as late hypocalcemia.

Recurrence status was confirmed through electronic records, and recurrence rates were calculated.

### 4.5. Ultrasonographic and Histopathological Volume Assessment

All patients underwent high-resolution neck ultrasonography in the preoperative period, and the maximum diameters of the adenoma in three orthogonal axes (cm) were recorded. Ultrasonographic volume was calculated in milliliters (mL) using the ellipsoid formula, which is widely used in clinical practice for approximating the volume of irregularly shaped structures based on three orthogonal dimensions:

V = π/6 × A × B × C

Ultrasonographic volume represents the estimated preoperative adenoma burden.

Excised parathyroid adenomas were measured macroscopically in the pathology laboratory, and three-dimensional dimensions were recorded in centimeters (cm). Histopathological volume was calculated in milliliters (mL) and was accepted as the reference indicator of true adenoma burden.

Both ultrasonographic and histopathological volume measurements were included in the analysis. Ultrasonographic volume was used as a preoperative estimate of adenoma size, while histopathological volume was accepted as the reference standard. The inclusion of both measurements enabled comparative evaluation.

Due to the retrospective design and long study period, ultrasonographic examinations and laboratory measurements were performed using the routine hospital imaging and laboratory systems in use at the time of patient evaluation. Detailed device information was not consistently available for all cases.

### 4.6. Statistical Analysis

Statistical analyses were performed using SPSS version 22.0 (IBM Corp., Chicago, IL, USA). The distribution of continuous variables was assessed using the Shapiro–Wilk test. Parametric tests were applied to normally distributed variables, while non-parametric tests were used for non-normally distributed data. Accordingly, Pearson correlation was used for normally distributed variables, and Spearman correlation was applied for non-normally distributed variables.

The relationships between preoperative biochemical parameters and ultrasonographic and histopathological adenoma volumes were evaluated using Pearson or Spearman correlation analyses. Correlation coefficients (r or ρ) were interpreted within the range of −1 to +1, and *p* < 0.05 was considered statistically significant.

Comparisons between surgical indication groups were performed using one-way ANOVA or the Kruskal–Wallis test, depending on data distribution.

Temporal changes in preoperative and postoperative biochemical parameters were analyzed using appropriate paired tests (paired *t*-test or Wilcoxon signed-rank test).

Categorical variables were presented as numbers and percentages (%), and complication and recurrence rates were calculated using descriptive statistics. Since follow-up duration was not normally distributed, it was expressed as median and interquartile range (IQR).

## 5. Conclusions

This large cohort study demonstrates a significant positive association between corrected serum calcium and parathyroid adenoma volume, particularly when measured histopathologically. Consistent with the literature, serum calcium levels generally parallel adenoma size, although this relationship is not absolute.

These findings suggest that, in primary hyperparathyroidism, the relationship between adenoma size and biochemical activity may be more reliably assessed through serum calcium levels. Further large-scale prospective studies are required to confirm these findings.

## Figures and Tables

**Table 1:** Baseline Demographic, Clinical, and Preoperative Biochemical Characteristics of the Study Cohort.

Variable	Total Cohort (n = 526)
Age (years), mean ± SD	52.44 ± 13.86
Gender, n (%)	
Female	434 (82.5)
Male	92 (17.5)
BMI (kg/m^2^), mean ± SD	26.25 ± 1.02
ASA Score, n (%)	
ASA I	73 (13.9)
ASA II	397 (75.5)
ASA III	55 (10.5)
ASA IV	1 (0.2)
Charlson comorbidity index, median (IQR)	2 (0–3)
Preoperative laboratory values ± SD	
Calcium (mg/dL)	11.26 ± 0.72
Albumin (g/dL)	4.21 ± 0.47
PTH (pg/mL)	198.49 ± 319.50
Phosphorus (mg/dL)	2.63 ± 0.82
25-OH Vitamin D (ng/mL)	21.18 ± 11.65
ALP (U/L)	118.99 ± 42.58

Values are presented as mean ± standard deviation (SD) unless otherwise indicated. The Charlson Comorbidity Index is presented as median and interquartile range (IQR). BMI: Body Mass Index; ASA: American Society of Anesthesiologists physical status classification; PTH: Parathyroid hormone; ALP: Alkaline phosphatase.

**Table 2:** Comparison of Postoperative Biochemical Parameters and Adenoma Volumes According to Surgical Indication.

Characteristic	Ureterolithiasis (n = 59)	Pathological Fracture (n = 8)	Musculoskeletal Symptoms (n = 228)	Hypercalcemia (n = 195)	Osteoporosis (n = 11)	Age < 50 Years (n = 24)	*p*-Value
Post-op Calcium (mg/dL), mean ± SD	9.72 ± 0.72	9.40 ± 0.00	9.40 ± 0.84	9.42 ± 0.92	9.70 ± 0.00	9.45 ± 0.15	0.205
Post-op PTH (pg/mL), mean ± SD	12.46 ± 12.10	7.70 ± 0.00	22.29 ± 34.03	20.63 ± 19.03	31.17 ± 16.87	14.25 ± 3.15	0.044
USG Volume (mL), mean ± SD	0.44 ± 0.39	2.06 ± 0.00	4.86 ± 35.65	0.53 ± 0.92	0.33 ± 0.69	1.36 ± 1.89	0.516
Pathology Volume (mL), mean ± SD	2.24 ± 3.43	1.72 ± 0.00	2.73 ± 4.55	0.84 ± 0.76	0.32 ± 0.21	2.29 ± 1.76	<0.001

Values are presented as mean ± standard deviation (SD). *p*-values were calculated using appropriate parametric or non-parametric multiple group comparison tests according to data distribution. USG: Ultrasonography; PTH: Parathyroid hormone. Surgical indications were defined as follows: ureterolithiasis, pathological fracture, and musculoskeletal symptoms were classified as symptomatic indications; hypercalcemia (serum calcium ≥ 1 mg/dL above the upper normal limit), osteoporosis (DXA T-score ≤ −2.5), and age < 50 years were considered guideline-based asymptomatic indications. No patient was operated solely due to renal involvement (glomerular filtration rate < 60 mL/min).

**Table 3:** Postoperative Complications, Follow-up Duration, and Recurrence Outcomes.

Variable	n	%
Early Postoperative Complications (≤1 month)		
No complication	382	80.8
Hypocalcemia	67	14.2
Reoperation due to bleeding	7	1.5
Vocal cord paralysis	8	1.7
Hypoparathyroidism	8	1.7
Surgical site infection	1	0.2
Mortality	0	0
Late Biochemical Outcome (6 months)		
Hypocalcemia at 6 months	9	2.8 *
Persistent hypocalcemia among early hypocalcemia cases	0	0
Follow-up and Recurrence		
Median follow-up duration (months)	8	(IQR 2–35)
Recurrence	7	1.45 †
No recurrence	475	98.55

* Calculated among patients with available 6-month calcium data (n = 318). † Calculated among patients with known recurrence status (n = 482).

## Data Availability

The data that support the findings of this study are available from the corresponding author upon reasonable request. The data are not publicly available due to privacy and ethical restrictions.
